# Intensified use of TAI and sexed semen on commercial farms

**DOI:** 10.21451/1984-3143-AR2018-0070

**Published:** 2018-09-06

**Authors:** Márcio de Oliveira Marques, Fábio Morotti, Elis Lorenzetti, Camila Bizarro-Silva, Marcelo Marcondes Seneda

**Affiliations:** 1 Geraembryo, Cornélio Procópio, Parana, Brazil.; 2 Laboratório de Reprodução Animal, DCV-CCA-UEL, Londrina, Parana, Brazil.; 3 Universidade Norte do Paraná, UNOPAR, Arapongas, Parana, Brazil.

**Keywords:** bull, conception rate, sex-sorted, pregnancy, timed artificial insjkemination

## Abstract

The livestock sector has achieved many technological advances, which have resulted in continued improvements in animal production systems and in the reproductive efficiency of herds. The associated use of reproductive biotechnology and genetic improvements combined with adequate sanitary and nutritional management are essential conditions for sustainable intensified animal production and financial autonomy within farms. Timed artificial insemination (TAI) represents one of the strategies with the greatest impact of expansion in providing genetic improvements and increased reproductive efficiency at a decreased cost. Despite the high proportion of cows receiving TAI, this market still exhibits considerable potential for expansion. After a TAI procedure, approximately 40 to 60% of females become pregnant. This result can vary depending on such factors as the hormonal protocol employed, female category, body condition score, ovarian status, farm management and aspects related to bulls and semen. The fertility and genetic quality of the bull plays an important role in the herd because a single bull can influence the entire production system. Another important strategy is the use of sex-sorted semen associated with TAI, primarily when associated with management practices to improve the pregnancy rate. This paper presents a review of the intensification of TAI, supplying practical information regarding the implementation of TAI commercial programs.

## Introduction

In the last two decades, the reproductive efficiency of cattle herds have achieved high success indexes due to the efforts of many studies focused on the development and improvement of reproductive biotechnologies, such as timed artificial insemination (TAI), superovulation and embryo transfer, as well as *in vitro* embryo production. In addition to reproductive techniques, genetic improvement programs have contributed greatly to increase livestock production, since these programs aim to select animals with the highest production merit. In this context, the combined use of reproductive biotechnologies with animals of high genetic quality has provided effective gains in both the quantity and the quality of livestock production systems.

Currently, the Brazilian cattle herd is composed of 218.23 million head (Instituto Brasileiro de Geografia e Estatística - IBGE, 2016 ). Roughly 83 million are reproductive-age cows, of which 26 million are intended for milk production, and approximately 57 million females are beef cattle (Associação Brasileira de Inseminação Artificial - ASBIA, 2017 ). Despite this favorable scenario, artificial insemination (AI) is used in only 10% of beef females, which highlights this biotechnology’s great potential for expansion. Conversely, of the approximately 12 million insemination procedures performed annually ( ASBIA, 2017 ), 85% of all artificially inseminated cows undergo TAI ( Baruselli *et al*., 2017b ). Certainly, the extensive use of TAI in comparison to conventional AI is related to the practicality of management and the increase of reproductive efficiency, since the fixed-time technique enables the insemination of a large number of cows without requiring estrus detection.

For a sustainable production system, the reproductive efficiency in both beef and dairy cattle is important. Thus, it is important to consider the number of calves produced, the genetic progress of the herd and the shorter interval between generations. To this end, we aim to present strategies that may be associated with the intensification of TAI management aimed at improving the reproductive efficiency and the productive sustainability of the farm.

## Factors influencing the conception rate in TAI

The intensification of TAI is related to the reproductive and economic optimization of a herd, as well as the objectives of each property ( Sá Filho *et al*., 2014 ). Moreover, the use of ovulation synchronization protocols enables the initiation of reproductive management in the early postpartum period, resulting in satisfactory and predictable rates when applied at properties with adequate nutritional and sanitary management. The breeding season allows a concentrated reproductive period and achiement of conception rates higher than 80% ( Marques *et al*., 2015 ). However, the exogenous control of follicular and luteal phases is fundamental because it permits the planning of programs without the need for estrus detection, and that associated with the early diagnosis of pregnancy can increase the reproductive performance of cattle ( Vasconcelos *et al*., 2006 ; Pugliesi *et al*., 2017 ).

**Figure 1:**
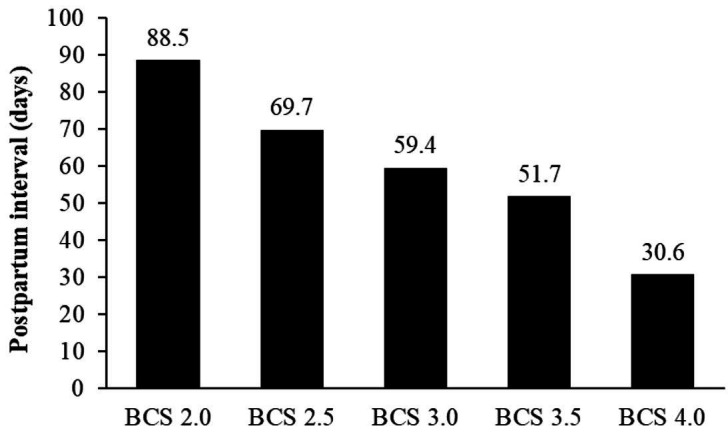
Influence of the body condition score (BCS) on the return of cyclicity in bovine females. Adapted from Houghton *et al*. (1990) .

The programmed breeding season is an important strategy for reproductive management in cattle. In practice, in beef cattle, the programmed breeding season aims to concentrate pregnancies and the calving season in favorable climatic conditions with greater pasture availability for matrices and calves ( Sá Filho *et al*., 2013 ). The immediate benefits of the programmed breeding season are related to optimization of hand labor, insemination of suckling females, organization of reproductive seasons, genetic improvements, reduction of the calving interval, and concentration of parturitions and weaning lots. As such, the body condition score (BCS) is a highly important tool for helping practitioners establish an optimal TAI program ( Fig. 1 ).

**Figure 2:**
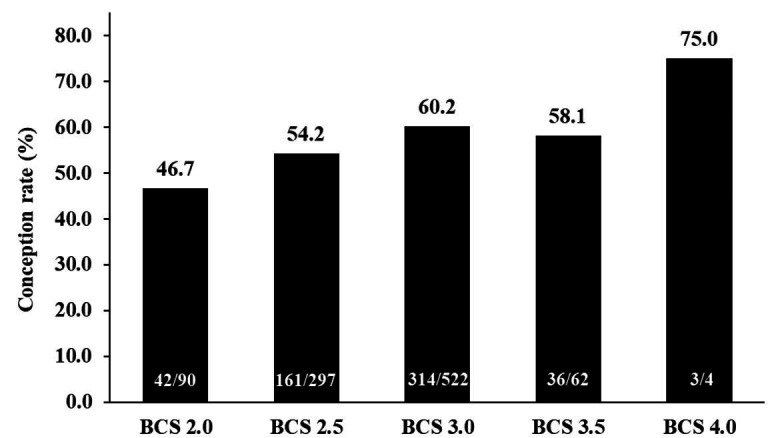
Effect of body condition score (BCS) on the pregnancy rate in cows that underwent timed artificial insemination (TAI) in the 2016/2017 breeding season (Marques *et al* ., 2018; Geraembryo, Cornélio Procópio, Parana, Brazil; unpublished data).

Variation in the BCS can generate a series of changes in the reproductive performance of cows. A low BCS can be associated with a high number of services per conception, increased intervals between calving and high percentage of non-pregnant cows ( Ferreira *et al*., 2013 ). Figure 2 shows the pregnancy rate of cows subjected to TAI program according to BCS.

Currently, pharmacological protocols for ovulation synchronization have reached a level of high efficiency for most situations on the farm. However, small adjustments according to the category of female (heifers, primiparous or multiparous; Table 1 ), BSC, postpartum period, ovarian status and subspecies (*Bos taurus* or *Bos indicus*) may be necessary for greater efficiency with TAI. Recently, Baruselli *et al*. (2017b) reviewed a large number of studies that used several TAI protocols with 3 or 4 handlings in which the source of progesterone (P4) was maintained for 8 or 9 days and concluded that in all situations, the pregnancy rate was similar. The choice of ovulation synchronization protocol should be based on the rational use of hand labor, promoting efficient management from both the reproductive and logistic perspectives of the farm ( Marques *et al*., 2015 ).

**Table 1:** Conception rates of Nelore heifers, primiparous and multiparous lactating cows after the first and second TAI (resynchronization).

Category	First TAI % (n/N)	Second TAI % (n/N)	First + Second TAI % (n/N)
Heifers	57 (514/903)	66^a^ (256/389)	85^a^ (770/903)
Primiparous	51 (173/338)	51^b^ (84/165)	76^b^ (257/338)
Multiparous	56 (680/1,223)	51^b^ (278/543)	78^b^ (958/1,223)

Adapted from Marques *et al*. (2015) .

**Figure 3:**
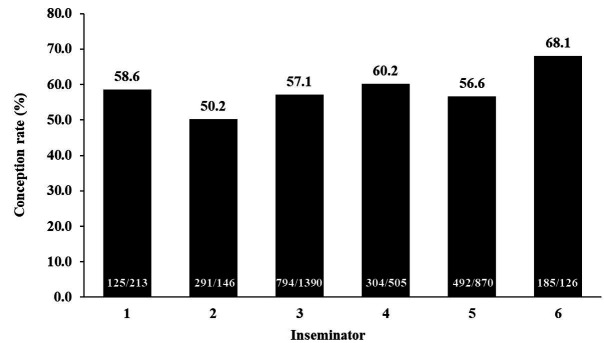
Effect of inseminator on the pregnancy rate after timed artificial insemination (TAI) in the 2006 breeding season (Marques *et al*., 2018; Geraembryo, Cornélio Procópio, Parana, Brazil; unpublished data).

To maintain a 365-day interval between calving and to improve reproductive efficiency, it is necessary to manage the reproductive records properly and to maintain the organization of the farm. The collection of the data for control of animals should begin with the calves’ birth and to continue throughout the productive life. During the breeding season, intensification in maintaining accurate records of the animals age, breed, category, BCS, postpartum period, protocol used, device loss, semen data, and inseminator ( Fig. 3 ) enables better management of reproductive rates and detection of possible failures ( Smith *et al*., 2012 ).

## Impact of the bull used for TAI

**Figure 4:**
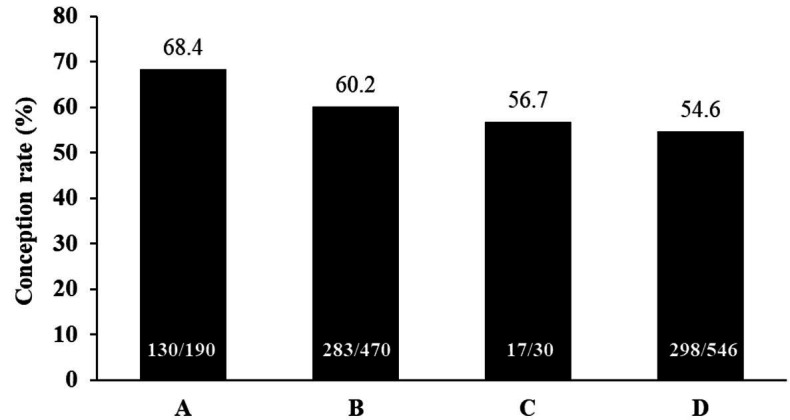
Conception rate in suckling Nelore cows (*Bos indicus*) using different Angus bulls for TAI during the 2016/2017 breeding season. Rates followed by different letters (^a or b^) were significantly different (P ≤ 0.05) (Marques *et al*., 2018; Geraembryo, Cornélio Procópio, Parana, Brazil; unpublished data).

The right selection of a bull according to the zootechnical value can add considerable genetic gains to the productive and reproductive efficiency of the herd. The bull can determine many characteristics in the progeny, such as weight at birth and weaning, age at puberty, weight gain, meat quality, milk production, probability of conception, age at first calving, and calving ease. Often, the choice of the bull is an accessible selection criterion with high repeatability and high heritability of reproductive biological potential ( Fonseca *et al*., 1992 ). It is important to note that there is great variability in individual fertility among bulls, even those with high genetic value and certificate of breeding soundness, and this difference in fertility may determine higher or lower reproductive performance in herds. For example, field studies conducted by our team with TAI in suckling Nelore cows identified the individual influence of bull fertility on conception rate ( Fig. 4 ) and weight at weaning ( Table 2 ).

**Table 2:** Weight at weaning and weight difference in the male calf (Angus x Nelore), using different Angus bulls for TAI during the 2015 breeding season.

Bull	N° animals (n)	Weight at weaning (kg)	Weight difference (kg)
A	36	256.4	+15.0
B	36	243.5	+2.0
C	32	242.4	0

(Marques *et al*., 2018; Geraembryo, Cornélio Procópio, Parana, Brazil;unpublished data).

## Use of sexed semen in TAI

The sex of the offspring is considered a determinant factor for productive and economic performance in both beef and dairy cattle. The male calf classically has little or no zootechnical value compared to the female calf in dairy cattle. Conversely, in beef cattle, the male calf is more valuable due to its greater potential for production and consequently higher aggregate economic value. In this context, many studies have focused on strategies to promote the birth of offspring of the desired sex and consequently increase the efficiency of production systems ( Pontes *et al*., 2010 ; Morotti *et al*., 2014 ; Pellegrino *et al*., 2016 ). Due to the discard of a large number of spermatozoids and due to the slow processing speed, the doses of sexed semen are commercialized with approximately 2 x 10^6^ of spermatozoa/straw ( Garner, 2006 ; Schenk *et al*., 2009 ).

The overall conception rate with sexed semen tends to be 50 to 60% of the rates found with conventional semen in cows and 70 to 90% in heifers ( Seidel *et al*., 1999 ; Seidel and Schenk, 2008 ; Butler *et al*., 2014 ). Moreover, even in heifers, conception tends to decrease with an increase in the number of services (47% for the 1st, 39% for the 2nd and 32% for the 3rd service; DeJarnette *et al*., 2009 ). Recently, seminal sexing has been performed based on next-generation technologies, for example, the use of SexedULTRA^TM^ (Sexing Technologies, Navasota, TX), which provides sex-sorted semen that is commercially available for dairy and beef cattle ( Thomas *et al*., 2017 ; Vishwanath and Moreno, 2018 ). With this semen type, adjustments in the composition of the medium that include the pre-staining seminal treatment, modifications in the staining medium itself and the freezing extenders contribute to greater balance and pH maintenance for prolonged times. Moreover, this sex-sorted semen is presented at a concentration of 4 x 10^6^ spermatozoa per straw ( de Graaf *et al*., 2014 ; Thomas *et al*., 2017 ; Vishwanath and Moreno, 2018 ).

**Figure 5:**
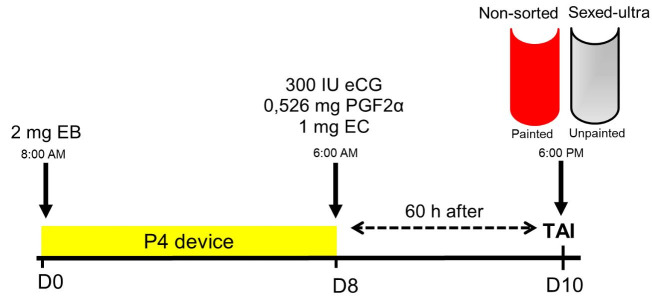
Scheme of TAI protocol performed in suckled Nelore cows inseminated with Sexed-ultra (cows unpainted at the base of the tail / estrus expression) or conventional semen/Non-sorted (cows painted at the base of the tail/no estrus expression) during the 2017 breeding season (Marques *et al*., 2018; Geraembryo, Cornélio Procópio, Parana, Brazil; unpublished data).

In Brazil, using this alternative of sex-sorted semen in beef cattle, Baruselli *et al*. (2017a) performed two studies in suckled Nelore cows that received the same TAI protocol for insemination with conventional semen *vs.* the different methodology of sex-sorted semen from three Nelore (sex-sorted for female, study I, n = 796) or three Angus bulls (sex-sorted for male, study II, n = 613). The cows were subjected a similar protocol ( Fig. 5 ); however, the cows’ handling was performed at the end of the day (5:00 to 7:00 pm), and TAI was accomplished on the morning of day 11 (5:00 to 7:00 am; 60 h after P4 device removal). In study I, there was no difference (P > 0.05) in the pregnancy rate among bulls, farms or interaction. However, there was a difference (P < 0.0001) in pregnancy rate for TAI according to the semen methodology used ( Table 3 ). In study II, the pregnancy rate was similar between the groups (P = 0.10). Although an effect of the farm was observed (P = 0.03), there was no difference in the pregnancy rate between bulls (P > 0.05), and no interaction was observed (P > 0.05).

**Table 3:** Pregnancy rate in suckled Nelore (n = 1,409) subjected to TAI and inseminated with conventional semen or different methodologies of sex-sorted semen.

Studies	Groups	Methodology	Pregnancy rate % (n/N)
I Nelore bulls	Conventional semen (20 x 10^6^ sptz)	Frozen semen without sexing	52.0^a^ (112/199)
	Sex-sorted (2,1 x 10^6^ sptz)	Previous sexing methodology	28.2^c^ (58/206)
	Sex-ultra 2 (2,1 x 10^6^ sptz)	Current sexing methodology	37.6^bc^ (72/191)
	Sex-ultra 4 (4 x 10^6^ sptz)	Current sexing methodology with enhanced concentration	43.0^b^ (86/200)
			
II Angus bulls	Conventional semen (20 x 10^6^ sptz)	Frozen semen without sexing	51.2 (107/209)
	Sex-ultra (4 x 10^6^ sptz)	Current sexing methodology with enhanced concentration	37.6 (84/200)
	Sex-ultra pure (4 x 10^6^ sptz)	Current sexing methodology with enhanced concentration (withbremoval of dead sptz)	43.0 (88/204)

Adapted from Baruselli *et al*. (2017a) .

**Figure 6:**
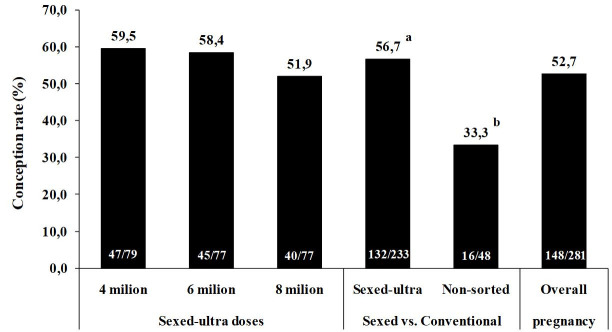
Conception rate in multiparous Nelore cows subjected to a timed artificial insemination (TAI) protocol with different Sexed-ultra doses of semen (cow in estrus) or conventional semen (no estrus) during the 2017 breeding season (Marques *et al*., 2018; Geraembryo, Cornélio Procópio, Parana, Brazil; unpublished data). Values denoted using lowercase letters ^(a-b)^ were different between Sexed-ultra and conventional semen. No difference (P = 0.320) was observed among Sexed-ultra doses).

A recent study evaluated the strategy of using sexed semen in a commercial application for TAI in beef cattle. Using Sex-ultra semen (current sexing methodology with enhanced concentration) with 4, 6 and 8 x 10^6^ sperm per dose from a single Aberdeen Angus bull, Nelore cows (n = 281) were submitted to TAI (Marques *et al*., 2018; Geraembryo, Cornélio Procópio, Parana, Brazil; unpublished data) according to the protocol depicted in Fig. 5 . As shown in Fig. 6 , no differences were found in the pregnancy rates when different concentrations of spermatozoa per dose were employed.

Interestingly, the data cited in Fig. 6 with sexed semen highlights a strategy with high efficiency for the use of sex-sorted semen in commercial field conditions. As such, we emphasize the positive results achieved (pregnancy rate higher than 50%), which can be attributed to the new methodology of sexed semen and also to the fact that this semen was only used in cows that expressed estrus, as indicated by unpainted tails.

## Sanitary control of gestational losses in TAI programs

Gestational losses in TAI programs may occur due to a variety of factors, such as genetics (e.g., anomalies), nutrition (e.g. deficiency and/or excess of macro or microelements), and zootechnological and health handlings ( Junqueira and Alfieri, 2006 ). While non-infectious causes are the main reason for reproductive losses in beef cattle, sanitary management is highly important because infectious diseases can cause pregnancy failures leading to direct or indirect economic losses ( Aono *et al*., 2013 ; Alfieri and Alfieri, 2017 ; Zangirolamo *et al*., 2017 ). Approximately 37 to 57% of gestational losses in cattle are caused by infectious diseases ( Khodakaram-Tafi and Ikede, 2005 ; McEwan and Carman, 2005 ).

Aono *et al*. (2013) demonstrated that primiparous lactating Nelore cows vaccinated against BVD, BoHV-1, and *Leptospira spp.*, showed lower gestational losses compared to unvaccinated herds, highlighting the importance of the control of these agents for reproductive efficiency in Brazilian cow-calf operations. These authors also concluded that it is best to vaccinate cows against these pathogens when the two doses are administered before TAI to ensure maximum antibody response and optimal reproductive outcomes. Similar results were described for lactating dairy cows vaccinated against BVD, BoHV-1, and *Leptospira spp.* and submitted to AI ( Pereira *et al*., 2013 ).

Ferreira *et al*. (2016) reported that beef cows vaccinated against foot-and-mouth virus resulted in a 4-fold increase in gestational loss when vaccinated 30 days after TAI compared with 31 days before TAI. The authors suggested that these outcomes can be associated with inflammatory and acute-phase reactions elicited by the foot-and-mouth vaccine. Similarly, a greater gestational loss was observed in Nelore heifers with twin gestations compared to heifers presenting a single gestation ( Marques *et al*., 2017 ).

## Conclusions

The livestock system has achieved representative technological advances in recent decades. TAI in particular has facilitated dissemination of high quality genetics, thereby improving the reproductive performance of herds. For the efficient use of this reproductive biotechnology, it is important to emphasize systematic control of the factors that affect TAI, control of gestational losses and the choice of bulls based upon fertility and genetic performance. In addition, sex-sorted semen can be considered a well-established reproductive biotechnology. Finally, the rational use of all of the strategies described in this review can contribute to intensification of TAI on the farm and improve the productive and reproductive performance of cattle.
